# Queen honey bee (*Apis mellifera)* survival and colony performance after overwintering mated queens indoors

**DOI:** 10.1093/jee/toaf022

**Published:** 2025-06-19

**Authors:** Leslie A Holmes, Jeffery Kearns, Nicole McCormick, Emily Olson, Lynae Ovinge, Patricia Wolf Veiga, Renata B Labuschagne, Shelley E Hoover

**Affiliations:** Department of Biological Sciences, University of Lethbridge, Lethbridge, Alberta, Canada; Department of Biological Sciences, University of Lethbridge, Lethbridge, Alberta, Canada; Alberta Technology Transfer Program, Alberta Beekeepers Commission, Edmonton, Alberta, Canada; Alberta Technology Transfer Program, Alberta Beekeepers Commission, Edmonton, Alberta, Canada; Alberta Agriculture and Forestry, Lethbridge Research Centre, Lethbridge, Alberta, Canada; National Bee Diagnostic Center, Northwestern Polytechnic, Beaverlodge, Alberta, Canada; Alberta Technology Transfer Program, Alberta Beekeepers Commission, Edmonton, Alberta, Canada; Department of Biological Sciences, University of Lethbridge, Lethbridge, Alberta, Canada

**Keywords:** queen banks, queen storage, queen overwintering, queen fertility

## Abstract

Honey bee, *Apis mellifera* L. (Hymenoptera: Apidae) winter colony mortality has reached sustained high levels and beekeepers depend on the availability of mated honey bee queens in early spring to recoup colony losses. Unfortunately, importing mated queens from other countries is currently the only reliable option meeting the demands of commercial beekeeping each spring in Canada. However, relying on queen imports brings another set of challenges, as supply chains can be disrupted, border crossings closed, imports prohibited, and the transportation of live animals can be stressful. This study explored the potential for Canadian beekeepers to supply queens in early spring by overwintering queens in queen banks that mated the previous summer. Queens were overwintered indoors in five queen banks. The following spring, the overwintered banked queens and a group of newly mated imported queens were introduced to colonies to evaluate queen introduction success and colony performance and survival over the following year. Queen survival in overwintered queen banks was low, with only 15% queen survival overall. Sperm viability of the banked queens prior to overwintering in queen banks was 30% higher than queens post-overwintering in queen banks. However, queen introduction success in the spring 2021, colony size, honey yield, and winter survival did not differ among queens that overwintered in queen banks and newly mated queens that were imported that spring. These results suggest banked overwintered queens have comparable performance to newly mated imported queens; although, overwintering mated queens in queen banks is risky, as entire queen banks can be lost, significantly reducing queen survival and the availability of mated queens in early spring.

## Introduction

The apiculture industry in Canada continues to grow with increasing demand for hive products and pollination services. With nearly 800,000 honey bee, *Apis mellifera L.* (Hymenoptera: Apidae) colonies among the 15,000 beekeepers in Canada in 2023, the country produced over 90 million pounds of honey valued at 277 billion CAD (Statistics Canada 2023). Honey bees also provide pollination services to several crops with economics benefits estimated at over $7 billion CAD (AAFC 2023). Each honey bee colony is normally headed by a single female, the queen. Shortly after emerging as adults, new queens take mating flights to mate with multiple males (ie drones) to acquire and store spermatozoa for the remainder of their lives, spending most of her remaining life inside the colony producing worker offspring (Winston 1987). The survival and productivity of the colony is therefore dependent on the presence of a healthy, fecund queen. Importantly, the economic benefits of the beekeeping industry in temperate climates, such as beekeeping regions of Canada but also other temperate climate regions including the northern USA and southern Chile, are specifically reliant on the availability of mated queen honey bees in early spring.

To ensure colony growth and productivity meet the demands of summertime pollination and honey production, spring marks a time when Canadian beekeepers assess colony mortality and queenlessness. With winter colony losses reaching up to 50% annually (CAPA 2022), beekeepers must rebuild their stock each spring by making new nucleus colonies and re-queening surviving queenless colonies as early as possible. However, production of newly mated queens in early spring in Canada is limited due to weather conditions and forage availability. While producing spring queens domestically is possible under ideal conditions in parts of Canada, the most reliable option to meet the demands of commercial beekeeping each spring is to purchase mated queens imported from other countries (eg USA, specifically California and Hawaii, New Zealand, Australia, Italy, Malta, and Chile). In fact, commercial beekeepers in Canada import more than 260,000 queens annually (AAFC 2023).

One challenge in relying on imported queens to rebuild colony losses in the spring is that the supply of imported queens can be halted for various reasons, as seen in 2020 when flights to Canada were canceled and/or delayed during COVID-19. In addition, importing queens from other countries may pose a risk of introducing diseases, pests, and unwanted genetics (CFIA 2013) (eg antibiotic resistant American foulbrood, small hive beetle, and *Tropilaelaps mercedesae* which is native to Asia, but establishment in Europe has been reported [Brandorf et al. 2024]). There is also the expense of importing queens and the potential stress transportation has on queens including temperature stress on queen fertility and viability (Pettis et al. 2016, McAfee et al. 2020, Rousseau et al. 2020, Holmes et al. 2023). Thus, finding strategies to supply Canadian beekeepers with queen honey bees in the early spring without relying on spring imports of queens have become an important ongoing research endeavor.

One strategy that has been explored is the mass storage of mated queens over winter (Wyborn et al. 1993, Gençer 2003, Rousseau and Giovenazzo 2021). Mated queens, even those that are imported, are often stored in the spring and summer in queenless honey bee colonies (herein referred to as queen banks) until they are needed. Queen banks are typically stocked with an abundance of nurse bees that care for dozens of individually caged queens for up to 3 mo (Reid 1975). However, banking queens for extended periods over winter may present challenges, specifically when temperatures drop in the winter and honey bees must cluster to maintain warmer temperatures. If the cluster of bees is not large enough to care for all the queens or moves elsewhere in the hive to seek out food stores, queens will not survive. In addition, the lack of winter brood rearing means that no new young nurse bees are being produced. Previous studies have focused on the survival and fertility of overwintered banked queens; however, colony performance and productivity of overwintered banked queens have not been characterized to date.

The objectives of this study were to provide proof of concept that queens could be overwintered in banks in our region, as per previously published studies elsewhere (Wyborn et al. 1993, Gençer 2003, Rousseau and Giovenazzo 2021), to assess the survival of queens in banks overwinter, and compare their performance the following season to that of newly imported queens. Thus, in this study, we overwintered queens that had mated in late summer in two-brood box queen banks. Queen banks were kept in indoor overwintering facilities from October 2020 to early April 2021. We measured survival of queens that were overwintered in queen banks and measured queen fertility on a subset of queens in queen banks prior to and after overwintering to determine whether overwintering queens in queen banks is a viable strategy to supply Canadian beekeepers with honey bee queens in the spring. In May 2021, banked overwintered queens and a group of newly mated imported queens were introduced to single-brood box (9-frame) colonies. We measured colony performance parameters, including brood pattern, honey production, and capped brood population size to evaluate performance of queens that overwintered in queens banks alongside newly mated queens imported in the spring.

## Materials and Methods

### Queen Sources

A total of 178 summer-mated queens, a combination of self-raised and purchased, were used in this experiment. In an apiary located near Lethbridge, Alberta, Canada, larvae (a mix of half and super-sisters) were grafted in late July 2020 to produce new queens. In late August, 36 of these queens were collected, marked with the year color, and caged in California mini cages in preparation for queen banking. In addition, in Edmonton, Alberta, 86 newly mated queens were purchased from local queen producers and 56 from California, USA; 36 of the California queens were sent to Lethbridge for overwinter banking.

### Overwinter Queen Bank Preparation

Two queen banks were overwintered indoors near Lethbridge and 3 queen banks were overwintered indoors near Edmonton. All queen banks were established in early September. In Lethbridge, the first queen bank housed the 36 queens produced from grafting, while the second queen bank housed the 36 California queens received from Edmonton. In Edmonton, 86 locally mated queens and 20 California queens were randomly assigned to the 3 queen banks.

To prepare the queen banks to house queens over winter, 2 strong queen-right double brood box colonies were dequeened and combined to produce each queen bank. Throughout September, each queen bank was supplemented with brood frames from other colonies in the apiary to continue brood production and maintain queen bank strength. However, by the end of September brood frames were not found in the apiary, suggesting queens had stopped laying. Each resulting four-box queen bank was provided pollen patties (1 in Edmonton, 2 in Lethbridge) and a total of 24L of sugar syrup (2:1 sugar:water) in a hive top feeder between August and October. In addition, queen banks were treated with ApiVar following label instructions to control *Varroa* mites.

Two honey frames per queen bank were modified to hold the caged queens. On one side of each frame, a rectangle (15 × 12.5 cm) was scraped down to the foundation near the center, creating space for the queen cages. Each frame held 18 queen cages taped together as per Rousseau and Giovenazzo (2021) arranged in a 9 × 2 block. These frames were placed in the top box of the colony, ensuring that the screened side of the queen cages faced each other with adequate bee-space for worker bees to access all queens.

In preparation for overwintering, each four-box queen bank was reduced to two-boxes in early October. The boxes containing the queen cages were placed on top of the heaviest other single box, and any light frames in the box housing the queens were replaced with full honey frames. All the workers bees were condensed into the 2 boxes, and the banks were overwintered in this configuration. The five queen banks were weighed and moved to their respective indoor winter storage facility in mid-October (ie one facility located near Edmonton, and one facility located near Lethbridge).

### Indoor Storage Conditions and Management

The 2 winter storage facilities near Edmonton and Lethbridge, respectively, had similar environmental conditions. Temperature was maintained at 15.06 ± 0.15 °C and 14.36 ± 0.11 °C in the indoor storage facilities in Lethbridge and Edmonton, respectively. Artificial lighting was not used apart from using red light during queen bank observations and data collection. Queen banks near Lethbridge were placed directly on weight scales in their indoor storage facility, allowing for monthly weight recordings of each queen bank throughout their overwintering. Queen banks overwintering near Edmonton were not placed on weight scales in their indoor winter storage facility and were therefore only weighed at the beginning and end of their overwintering.

### Queen Fertility Analyses

Prior to winter storage, 6 mated queens (ie 2 from each of the 3 queen banks in Edmonton) were sent to the National Bee Diagnostic Center (NBDC) in Beaverlodge, Alberta for queen fertility analysis in October 2020. In April 2021, five mated queens from the 2 surviving queen banks in Edmonton were also sent to the NBDC in Beaverlodge, Alberta for queen fertility analysis.

To determine queen fertility (ie sperm number and viability) of banked queens prior to winter and after overwintering, the abdomens of the 11 queens from the Edmonton location were dissected individually while being viewed through a stereomicroscope (Model SMZ1000, Nikon). Their spermathecas were removed and immediately ruptured in 0.5 ml of Buffer D (Collins and Donoghue 1999) in a glass vial. Spermatozoa were then stained with fluorescent markers (LIVE/DEAD Sperm Viability Kit, Molecular Probes, Eugene, OR, USA) following Holmes et al. (2023). Both live (fluoresces at green wavelengths [eg EM 520-570 nm]) and dead (fluoresces at red wavelengths [eg EM 650 nm]) sperm were visualized and counted using a fluorescence microscope (Fluoview FV10C-W3, Olympus, Tokyo, Japan). Average sperm count was corrected for dilution using a 0.02-mm Thoma counting chamber (Hawksley and Sons Ltd, Lancing, Sussex, UK).

### Removal from Indoor Winter Storage

The Lethbridge queen banks were moved from indoor storage to an apiary located at the University of Lethbridge in mid-March 2021and provided 2 pollen patties and 8 liters of sugar syrup (1:1 sugar:water) in frame feeders. The Edmonton queen banks were moved outdoors in early-April 2021; 2 of the 3 Edmonton queen banks were alive at this time. Edmonton queen banks were provided a pollen patty and 8 liters of sugar syrup.

### Colony Performance Parameters

After both queen banks that overwintered in Lethbridge were found dead in the spring, the surviving queens that overwintered in queen banks in Edmonton and the newly mated imported queens that arrived in Edmonton were randomly divided among the two apiary sites. Specifically, 11 banked overwintered queens and 22 newly mated imported queens (ie 11 Malta and 11 California) were introduced to 33 single brood box colonies in Edmonton, while 11 banked overwintered queens and 21 newly mated imported queens from Malta (*n* = 5) and California (*n* = 16), were sent to Lethbridge and introduced to 32 newly split single brood box colonies in early May. Performance of the queens was assessed throughout the 2021 season, as well as their overwintering success during the 2021 to 2022 winter. At each apiary, banked overwintered queens and newly mated imported queens (ie received early spring 2021) were introduced to single brood box colonies using a slow-release method where the exit was blocked with candy at research apiaries near Lethbridge and Edmonton. Four days post queen introduction, colonies were checked for queen status and queens still inside their cages were either direct released or assisted by removing some of the candy blocking the cage exit.

Colony performance and productivity parameters including varroa infestation level, brood pattern, honey yield, capped brood population size, and fall and spring cluster size were assessed for each colony at both sites. Colonies were monitored throughout the summer 2021 and any observations of brood disease and failing queens were recorded. Colonies were removed from the experiment if the original queen was no longer present.

Each colony was sampled for *Varroa* mites 3 times throughout the 2021 season and again in April 2022 following techniques of Gatien and Currie (2003), where approximately 300 bees were collected from brood nest frames. Specifically, colonies were sampled for *Varroa* mid-May 2021 and again in September and October, before and after a fall Thymovar (Lethbridge) or Formic Pro (Edmonton) treatment, respectively. Due to higher *Varroa* mite levels in colonies near Lethbridge after the October *Varroa* sampling, Lethbridge colonies were treated with oxalic acid fumigation following label instructions in November 2021.

Brood patterns were evaluated using a rhombus-shaped cutout of corrugated plastic encompassing 100 cells (10 × 10) overlaid on a patch of capped brood. Patches with the greatest contiguity of capped brood were selected, and 4 patches were evaluated per colony on at least 2 separate frames. Brood pattern solidness was calculated by subtracting the number of cells not containing capped brood from 100. Measurements of brood solidness were collected in June, July, and August 2021 and averaged for each colony prior to analysis.

Pre-weighed honey supers were added to each colony in mid-June 2021 and throughout the honey flow as needed. At the end of the honey flow, honey supers were removed, and their full weights recorded. Total honey production for each colony was calculated by subtracting the honey super pre-weights from the honey super full weights. After removing the honey supers, a second brood box was added to each colony to ensure they had adequate space and resources for overwintering.

The number of sealed worker brood was evaluated early- to mid-August 2021. Frames containing capped worker brood had the bees removed by shaking or brushing the bees into the brood box. Frames were then placed in a photo box where each side of the brood frame was photographed using a high-resolution camera. The number of sealed worker brood cells was analyzed using the HoneybeeComplete software (version 5.4 WSC Scientific, Heidelberg, Germany).

Bee cluster size was measured for each colony in fall 2021 and spring 2022 by visually estimating the number of inter-frame spaces filled with bees from both the top and bottom of each colony’s brood box (Nasr et al. 1990). The total number of inter-frame spaces from top and bottom views was used in the analysis.

### Statistical Analyses

All statistical analyses were done using the R software environment version 4.4.0. (R Core Team 2023).

The survival of banked overwintered queens in the 5 queen banks in Lethbridge and Edmonton was reported as the proportion of queens that survived until their introduction to assessment colonies in May 2021.

We performed Welch’s *t* tests to compare sperm count and sperm viability of the banked queens before (*n* = 6) and after (*n* = 5) overwintering. Sperm viability was estimated from the number of living and dead sperm and expressed as a proportion of viable sperm.

The survival of banked overwintered queens and newly mated imported queens introduced to assessment colonies was determined at 3 time points between spring 2021 and spring 2022: (i) introduction success was analyzed as the number of introduced queens that survived until the end of June 2021. We used a 42-d period to assess queen introductory success (ie the length of 2 brood cycles); (ii) season success was analyzed as the proportion of queens successfully introduced to colonies in May 2021 that survived until October 2021, when colonies were being prepared for winter; (iii) overwintering success was analyzed as the number of queens that survived overwintering relative to the number of queens alive in October 2021. To characterize these survival rates among the 3 queen stocks (ie banked overwintered, California imported, and Malta imported), we used generalized linear models (GLMs) with a binomial logit link error distribution, using the *mgcv* package (Wood 2004). Statistical significance was evaluated using model selection with Akaike Information Criteria (AIC) (Burnham and Anderson 2002, Wood et al. 2016, Wood 2017). The general linear hypothesis testing function *glht* in the Multcomp package (Hothorn et al. 2008) was used for post-hoc analyses of GLMs, where applicable; *P* values were adjusted for Type I error using the Bonferroni method.

Generalized linear models and AIC model selection were also used to characterize mean brood solidness score, the number of sealed brood, mean honey yields, and bee cluster sizes, across the categorical variable of queen stock source (ie banked overwintered, California imported, and Malta imported). In addition, mean *Varroa* mite levels were characterized across the 3 queen sources and location (ie Edmonton and Lethbridge) using generalized linear models and AIC model selection for each sampling time point (eg spring 2021, pre- miticide treatment, post- miticide treatment, and spring 2022).

## Results

### Overwintering Late Season Mated Queens in a Queen Bank

**Fig. 1.**
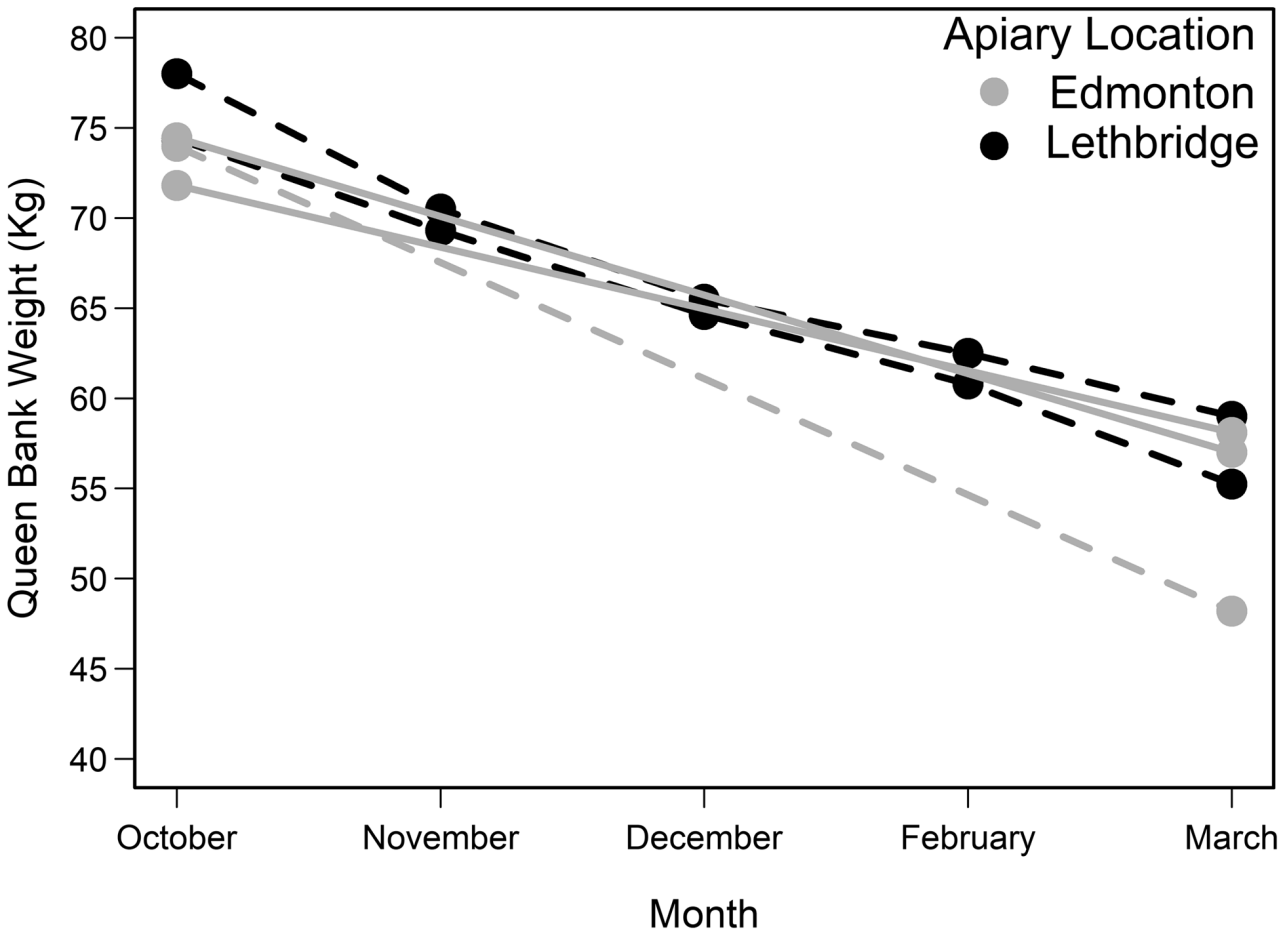
Queen bank weight (kg) throughout winter (ie October 2020 to March 2021). Queens (*n* = 172) were caged and overwintered in 5 two-brood-box queen banks. Three queen banks were located near Edmonton, AB (gray points and lines) and two queen banks were located near Lethbridge, AB (black points and lines). Queen bank weights with dashed lines are queen banks that did not survive overwintering, solid lines represent surviving queen banks.

Queens mated in late summer (ie August) (*n* = 172) were caged and overwintered in 5 two-brood-box queen banks (ie 3 queen banks in Edmonton and 2 queen banks in Lethbridge, Alberta). All 5 queen banks were similar in strength and weight prior to overwintering (Mean±SE=74.52±1.00   kg; χ=2.92;   df=3;   P=0.40) (Table 1). Queen bank weight loss over winter was similar across all 5 queen banks (Fig. 1). However, only 27 out of 172 queens survived winter banking (Mean±SE=   15.88±9.74   % ). Three of the 5 queen banks did not ultimately survive winter storage (Table 1). While the 2 Lethbridge queen banks were both alive when initially removed from the indoor winter storage facility in mid-March 2021, with 23.43 % of their queen remaining alive, both queen banks were found dead at the end of April 2021.

**Table 1.** Queen bank survival (%) and queen bank pre-winter and post-winter weights (kg). Queens were mated in late August 2020 and overwintered in strong two-brood box queen banks. Queen bank survival is calculated as of 1 May 2021. Queen banks were provided 2:1 sugar syrup and pollen patties in fall 2020 and 1:1 sugar syrup and pollen patties in spring 2021.

Queen bank	No. of queens banked	Percent survival	Pre-winter weight (kg)	Post-winter weight (kg)	Weight loss (kg)
Edmonton 1	32	0.0%	74.0	48.2	25.8
Edmonton 2	34	38.2%	74.4	57.0	17.4
Edmonton 3	34	41.2%	71.8	58.1	13.7
Lethbridge 1	36	0.0%	74.4	55.2	19.2
Lethbridge 2	36	0.0%	78.0	59.0	19.0

Sperm count and sperm viability were measured on a subset of queens prior to overwinter banking in October 2020 (*n* = 6), and after winter queen banking in April 2021 (*n* = 5). The average sperm count did not differ in the spring after overwinter banking compared to in the previous autumn Mean±SE=7.95±0.62   million   sperm;   t=0.04;   df=8.19;   P=0.97 There was, however, a significant decrease in the viability of sperm present in the spring after overwinter banking (Mean±SE=70.90±5.70 %), compared to the autumn prior to winter banking (Mean±SE=96.83±1.45   % ) (t=−4.41;   df=4.52;   P<0.01).

### Colony Performance of Banked Overwintered Queens and Newly Mated Imported Queens

Queen introduction success was evaluated in June 2021, after 2 brood cycles had passed, and did not significantly differ among queen sources. Specifically, 88.89%, 75.0%, and 63.64% of California, Malta, and banked queens, respectively, were successfully introduced to their assessment colonies (ANOVA: *F* = 2.25; df = 2; *P* = 0.114; Supplementary Table S1). However, by October 2021, the number of queens remaining in the project did differ among queen sources (ANOVA: *F* = 4.23; df = 2; *P* = 0.02; Supplementary Table S1). A greater percentage of successfully introduced banked overwintered queens (78.6%) were still heading colonies going into winter compared to successfully introduced newly imported queens from Malta (25.0%), with queens from California (58.3%) showing intermediate success. Winter colony mortality did not differ among queen sources in spring 2022 (ANOVA: *F* = 1.70; df = 2; *P* = 0.20; Supplementary Table S1). Survival rates were 78.57%, 100%, and 100% for newly imported queens from California and Malta, and banked overwintered queens, respectively, going into winter.

Colony performance parameters, including brood solidness score, the number of capped brood cells, honey yield, and cluster scores in fall 2021 and spring 2022, did not vary by queen source, but did vary by apiary location (ie Edmonton and Lethbridge), where brood solidness score, the number of capped brood cells, honey yield, and the spring 2022 cluster score were all significantly higher in colonies located near Edmonton than in the colonies located near Lethbridge (Table 2, Supplementary Table S2).

**Table 2.** Mean ± SE colony performance parameters measured for newly imported queens (ie California and Malta) that were mated in the early spring of 2021 and banked overwintered queens that were mated in August 2020 and overwintered in queen banks in Alberta. All queens were introduced to their assessment colonies in Edmonton and Lethbridge, Alberta in May 2021 and followed until April 2022. Means and standard errors of colony performance parameters that are significantly different across apiary locations (ie Edmonton and Lethbridge) are indicated by different superscript letters (*P* < 0.05) after performing general linear hypothesis post-hoc testing on the top model selected by Akaike Information Criterion (AIC) (Supplementary Table S2). No significant differences were found among queen sources for any colony performance parameter.

Queen source	Brood solidness score (%)	No. of capped brood (×1,000)	Fall 2021 cluster score	Spring 2022 cluster score	Honey yield (kg)
	*n* = 51	*n* = 32	*n* = 28	*n* = 25	*n* = 31
California	91.65 ± 1.24	11.19 ± 0.93	15.34 ± 0.51	5.88 ± 1.10	41.61 ± 6.15
Malta	95.26 ± 1.04	10.75 ± 2.33	14.88 ± 0.85	7.58 ± 3.46	36.04 ± 10.91
Banked Overwintered	91.49 ± 1.18	9.22 ± 1.32	14.74 ± 0.72	6.61 ± 1.10	30.68 ± 5.15
Apiary Location					
Edmonton	96.02 ± 0.66^a^	13.25 ± 0.90^a^	15.61 ± 0.72	8.81 ± 1.36^a^	54.05 ± 5.38^a^
Lethbridge	89.29 ± 0.94^b^	8.18 ± 0.77^b^	14.69 ± 0.45	5.27 ± 0.80^b^	22.34 ± 1.58^b^

Similarly, *Varroa* mite levels did not vary by queen source in the spring 2021 when queens were introduced, nor in the fall 2021 before and after miticide treatment (Supplementary Table S3). Instead, *Varroa* mite levels differed between the 2 apiary locations throughout the 2021 season (*t* = 7.07; df = 23; *P* < 0.01; Supplementary Table S3), with colonies near Edmonton (mean ± SE = 1.47 mites per bee) having higher *Varroa* mite levels than colonies near Lethbridge (mean ± 0.50 mites per bee). In spring 2022, *Varroa* mite levels exhibited an interaction between queen source and location (*t* = 2.88; df = 19; *P* < 0.01; Supplementary Table S3). However, after excluding the single Malta queen-led colony located near Edmonton with an average mite load of 7.74 mites per bee from the data, the top model included only location (Supplementary Table S3), with *Varroa* mite levels remaining higher in colonies near Edmonton (mean ± SE = 2.08 mites per bee) compared to colonies near Lethbridge (mean ± SE = 0.84 mites per bee).

## Discussion

In this study, at 2 separate locations in Alberta, Canada, queens that had mated in late summer were overwintered indoors in queen banks. Surviving banked overwintered queens were then introduced to assessment colonies the following spring along with newly mated imported queens from California, USA and Malta. We evaluated colony performance and productivity for the introduced banked overwintered queens and newly mated imported queens for a year. Our results suggest that when successfully introduced, banked overwintered queens perform well in their colonies. However, supplying Alberta beekeepers with banked overwintered queens in early spring as an alternative to relying on importing queens is a risky endeavor and may not be economically sustainable due to low overwinter survival and time investments.

Interestingly, the 2 queen banks in Lethbridge were alive in March 2021 with 44% and 2.86% queen survival in the 2 queen banks, respectively, although queen bank 2 had only a single surviving queen. However, by the end of April 2021, both queen banks were dead and abandoned. The loss of queen banks in Lethbridge between March and the end of April 2021 is unclear but may be related to the climate and apiary environment. March 2021 was mild with a mean outdoor temperature of 3.5 °C and extreme highs and lows near 15.2 °C and −11.4°C, respectively, near the end of March (Environment and Climate Change Canada 2024) after the queen banks were moved outdoors. April was colder than March on average, with a mean outdoor temperature of −3.7 °C and extreme highs and lows near 20.9 °C and −11 °C, respectively (Environment and Climate Change Canada 2024). There were days near the end of March with wind gusts above 100 km/h, but precipitation was minimal to nil; April had a total of 19 mm of precipitation with wind gusts of 55 km/h on average (Environment and Climate Change Canada 2024). Weather may not explain the queen bank losses, and it is worth noting that we observed evidence of small mammal predator damage (eg shrews) on the colonies that overwintered in the apiary the queen banks were moved to, and therefore, we cannot rule out predation as a factor of queen bank mortality and/or abandonment.

Despite using similar queen bank colony preparations to Rousseau and Giovenazzo (2021), and indoor overwintering temperatures above honey bee cluster formation found to be optimal for queen survival, our banked queen survival was only 15% overall, compared to 86% achieved by to Rousseau and Giovenazzo (2021) under similar conditions. Further, while Wyborn et al. (1993) found banked overwintered queen survival of 60% over 2 years (where queens were overwintered outdoors in queen banks), they too had a year in their 3-year study where banked queen survival was very low, suggesting overwintering banked queens may not be a reliable management practice on a commercial scale, or may be more suitable in certain climates.

The subset of banked queens that we performed fertility analysis on prior to and after overwintering had significantly lower sperm viability after overwintering. However, despite reduced sperm viability, these overwintered queens performed similarly to the newly mated imported queens in the field with respect to colony size, honey yield, and winter colony survival (Table 2). It is possible these overwintered queens with lower sperm viability may fail earlier in their second season; however, this would only be a concern for beekeepers who did not replace their queens annually. Interestingly, Rousseau and Giovenazzo (2021) and Levesque et al. (2023) did not find differences in sperm viability among queens banked overwinter compared to control queens that overwintered in their respective colonies for similar lengths of time, suggesting our banked overwintered queens may have encountered some additional stress during overwintering that impacted their fertility, which may or may not have also led to the high queen mortality we found in our banked overwintered queens. However, Rousseau and Giovenazzo (2021) did not report the fertility of banked queens and control queens prior to overwintering, making it difficult to interpret any impact the overwintering treatments (banked versus individually loose in colonies) may have had on queen fertility in their study.

Queen introduction success in spring 2021 did not differ among the banked overwintered queens and newly mated imported queens, where average queen introduction success was 75.84%. Our queen introduction success was 8% to 13% lower than other studies that recently overwintered banked queens in similar conditions (Rousseau and Giovenazzo 2021, Levesque et al. 2023). However, queen introduction success in these studies was evaluated 7 to 12 d post queen introduction (Rousseau and Giovenazzo 2021, Levesque et al. 2023) as opposed to 42 d post queen introduction in our study. Of the successfully introduced queens, the banked overwintered queens had 30% to 100% more of their queens survive until winter preparations in October 2021 compared to newly mated California and Malta queens, respectively, suggesting that despite successful queen introduction, queen retention throughout the summer and fall was a much greater challenge for the newly mated imported queens than the banked overwintered queens that mated in late summer the previous season.

While banked overwintered queens performed similarly to newly mated imported queens from California and Malta in the field, we did find significant differences in colony performance and productivity between the 2 apiary locations (ie Edmonton and Lethbridge, AB). Specifically, colonies located near Edmonton, AB were significantly larger, yielded more honey, and had higher levels of Varroa mite infestations; although these differences between the 2 locations are consistent with provincial trends in Alberta and other studies (Government of Alberta 2023, Holmes et al. 2023, Peirson et al. 2024). Specifically, Alberta’s north west region where Edmonton is located, consistently has larger colonies, honey yield, and higher Varroa mite levels than Alberta’s south region where Lethbridge is located (Alberta Tech Transfer Program 2023, Government of Alberta 2023, Holmes et al. 2023). These differences in colony performance and productivity between these regions of Alberta are likely attributed to the seasonal and annual differences in climate and nectar and pollen flows.

Lastly, despite using similar methodologies to other successful overwintering queen bank studies (Rousseau and Giovenazzo 2021, Levesque et al. 2023), our low queen survival rates of banked overwintered queens suggest that methodologies to overwintering queen banks in Canada may need to be adapted to the specific geographic location and beekeeping practices. For example, Québec (QC), British Columbia (BC), and Turkey, where banked queens have successfully overwintered indoors and outdoors (Wyborn et al. 1993, Gençer 2003, Rousseau and Giovenazzo 2021, Levesque et al. 2023), have drastically different winters than Alberta. Alberta has long, cold, and dry winters compared to shorter, mild, and humid winters in BC, QC, and Turkey. Alternatively, overwintering mated queens in small mating hives or nucleus colonies may prove more successful in Alberta than overwintering banked queens, as these strategies have shown to be more successful than overwintering banked queens in other studies (Wyborn et al. 1993, Gençer 2003).

## Supplementary Material

Supplementary material is available at *Journal of Economic Entomology* online.

toaf022_suppl_Supplementary_Tables_S1-S3

## Data Availability

The data used for this study is available from the corresponding authors on reasonable request.
