# Supplementary material for: Queen honey bee (Apis mellifera) survival and colony performance after overwintering mated queens indoors
Source: J Econ Entomol. 2025 Jun 19;118(4):1512–8. doi: 10.1093/jee/toaf022 (PMC12397964; doi:10.1093/jee/toaf022)
Supplement: toaf022_suppl_Supplementary_Tables_S1-S3 [file toaf022_suppl_supplementary_tables_s1-s3.docx]

**Supplementary Information**

Queen honey bee (*Apis mellifera*) survival and colony performance after overwintering mated queens indoors

L.A. Holmes, J. Kearns, N. McCormick, E. Olson, L. Ovinge, P.W. Wolf Veiga, R.B. Labuschagne, and S.E. Hoover.

**Table S1.** Mean ± SE survival of banked overwintered queens and newly mated imported queens at three time points between spring 2021 and spring 2022: (1) introduction success, Y; (2) season success, Y; (3) overwintering success, Y. Queens were introduced to assessment colonies in spring 2021. Generalized linear models with a binomial logit link error distribution and model selection with Akaike Information Criteria (AIC) were used to characterize the effect of queen source (Q) (i.e., California, Malta, and summer mated winter banked) on queen survivorship. Mean ± SE survival with different letters within a survival column (i.e., Introduction, season, or overwintering success) are significantly different, (p < 0.05) after performing general linear hypothesis post-hoc testing on the top model selected by Akaike Information Criterion (AIC).

| **Model** | **No. of Observations** | **ΔqAIC** | | **Df** | **Weight** | | **Residual Deviance** |
| --- | --- | --- | --- | --- | --- | --- | --- |
| **Introduction Success** |  |  | |  |  | |  |
| Y ~ Q | 65 | 0.00 | | 3 | 0.53 | | 65.67 |
| Y ~ 1 | 65 | 0.22 | | 1 | 0.47 | | 70.23 |
| **Season Success** |  |  | |  |  | |  |
| Y ~ Q | 50 | 0.00 | | 3 | 0.85 | | 60.65 |
| Y ~ 1 | 50 | 3.51 | | 1 | 0.15 | | 68.59 |
| **Overwintering Success** |  |  | |  |  | |  |
| Y ~ 1 | 28 | 0.00 | | 1 | 0.54 | | 19.07 |
| Y ~ Q | 28 | 0.33 | | 3 | 0.46 | | 14.55 |
| **Queen Source** | **Mean ± SE Survival (%)** | | | | | | |
|  | **Introduction Success** | | **Season Succes** | | | **Overwintered** | |
| **Newly Mated California** | 88.89 ± 6.16 (n = 27) ^a^ | | 58.33 ± 10.28 (n = 24) ^ab^ | | | 78.54 ± 11.38 (n = 14) ^a^ | |
| **Newly Mated Malta** | 75.00 ± 11.18 (n = 16) ^a^ | | 25.00 ± 13.05 (n = 12) ^a^ | | | 100.00 ± 0.00 (n = 3) ^a^ | |
| **Banked Overwintered** | 63.64 ± 10.50 (n = 22) ^a^ | | 78.57 ± 11.38 (n = 14) ^b^ | | | 100.00 ± 0.00 (n = 11) ^a^ | |

**Table S2.** Generalized linear models with their respective link error distributions and model selection with Akaike Information Criteria (AIC) was used to characterize the effect of queen source (Q) (i.e., California, Malta, and banked overwintered) and location, L, (i.e., Edmonton and Lethbridge) on brood solidness scores, Y, the number of capped brood, Y, fall 2021 cluster score, Y, spring 2022 cluster score, Y, and honey yield, Y.

| **Model** | **No. of Observations** | **ΔqAIC** | **Df** | **Weight** | **Residual Deviance** |
| --- | --- | --- | --- | --- | --- |
| **Brood Solidness Score** | | | | | |
| Y ~ L | 51 | 0.00 | 3 | 0.86 | 865.11 |
| Y ~ L + Q | 51 | 3.93 | 5 | 0.12 | 850.06 |
| Y ~ L * Q | 51 | 8.09 | 7 | 0.01 | 831.71 |
| Y ~ 1 | 51 | 23.70 | 2 | 0.00 | 1439.33 |
| Y ~ Q | 51 | 23.74 | 4 | 0.00 | 1315.60 |
| Error Distribution: Gaussian with identity link | |  |  |  |  |
| **No. of Capped Brood** | | | | | |
| Y ~ L | 32 | 0.00 | 3 | 0.79 | 32.75 |
| Y ~ L + Q | 32 | 3.02 | 5 | 0.18 | 32.70 |
| Y ~ L * Q | 32 | 7.55 | 7 | 0.02 | 32.67 |
| Y ~ 1 | 32 | 8.67 | 2 | 0.01 | 52.18 |
| Y ~ Q | 32 | 12.39 | 4 | 0.00 | 33.00 |
| Error Distribution: Negative Binomial with log link | |  |  |  |  |
| **Fall 2021 Cluster Score** | | | | | |
| Y ~ 1 | 28 | 0.00 | 2 | 0.58 | 118.33 |
| Y ~ L | 28 | 1.14 | 3 | 0.33 | 112.64 |
| Y ~ Q | 28 | 4.70 | 4 | 0.06 | 116.00 |
| Y ~ L + Q | 28 | 6.11 | 5 | 0.03 | 109.63 |
| Y ~ L * Q | 28 | 12.46 | 7 | 0.00 | 107.60 |
| Error Distribution: Gaussian with identity link | |  |  |  |  |
| **Spring 2022 Cluster Score** | | | | | |
| Y ~ L | 25 | 0.00 | 3 | 0.76 | 277.16 |
| Y ~ 1 | 25 | 2.90 | 2 | 0.18 | 345.35 |
| Y ~ L + Q | 25 | 5.59 | 5 | 0.05 | 272.50 |
| Y ~ Q | 25 | 7.79 | 4 | 0.02 | 337.62 |
| Y ~ L * Q | 25 | 12.78 | 7 | 0.00 | 269.94 |
| Error Distribution: Gaussian with identity link | |  |  |  |  |
| **Honey Yield** | | | | | |
| Y ~ L | 31 | 0.00 | 3 | 0.61 | 5957.30 |
| Y ~ L + Q | 31 | 2.19 | 5 | 0.20 | 5352.70 |
| Y ~ L * Q | 31 | 2.30 | 7 | 0.19 | 4359.80 |
| Y ~ 1 | 31 | 23.30 | 2 | 0.00 | 13676.90 |
| Y ~ Q | 31 | 26.55 | 4 | 0.00 | 12878.00 |
| Error Distribution: Gaussian with identity link | |  |  |  |  |

**Table S3.** Generalized linear models with a gaussian identity link error distribution and model selection with Akaike Information Criteria (AIC) was used to characterize the effect of queen source (Q) (i.e., California, Malta, and banked overwintered) and location, L, (i.e., Edmonton and Lethbridge) on *Varroa* mite levels in spring 2021, Y, fall 2021 before miticide treatment, Y, fall 2021 after miticide treatment, Y, spring 2022, and spring 2022 after removing the single Malta queen in Edmonton from the data, Y.

| **Model** | **No. of Observations** | **ΔqAIC** | **Df** | **Weight** | **Residual Deviance** |
| --- | --- | --- | --- | --- | --- |
| **Spring 2021** | | | | | |
| Y ~ 1 | 50 | 0.00 | 2 | 0.61 | 4.35 |
| Y ~ L | 50 | 2.22 | 3 | 0.20 | 4.34 |
| Y ~ Q | 50 | 2.97 | 4 | 0.14 | 4.20 |
| Y ~ L + Q | 50 | 5.28 | 5 | 0.04 | 4.19 |
| Y ~ L * Q | 50 | 10.32 | 7 | 0.00 | 4.17 |
| **Fall 2021 Before Miticide Treatment** | | | | | |
| Y ~ L | 30 | 0.00 | 3 | 0.39 | 42.23 |
| Y ~ L * Q | 30 | 0.19 | 7 | 0.36 | 28.33 |
| Y ~ L + Q | 30 | 0.93 | 5 | 0.25 | 36.17 |
| Y ~ 1 | 30 | 35.68 | 2 | 0.00 | 150.68 |
| Y ~ Q | 30 | 40.36 | 4 | 0.00 | 148.28 |
| **Fall 2021 After Miticide Treatment** | | | | | |
| Y ~ L | 29 | 0.00 | 3 | 0.46 | 25.22 |
| Y ~ 1 | 29 | 0.73 | 2 | 0.32 | 28.18 |
| Y ~ L + Q | 29 | 2.56 | 5 | 0.13 | 22.67 |
| Y ~ Q | 29 | 3.22 | 4 | 0.09 | 25.67 |
| Y ~ L * Q | 29 | 8.87 | 7 | 0.00 | 22.35 |
| **Spring 2022** | | | | | |
| Y ~ L * Q | 25 | 0.00 | 7 | 0.80 | 59.30 |
| Y ~ L | 25 | 3.63 | 3 | 0.13 | 79.83 |
| Y ~ L + Q | 25 | 5.31 | 5 | 0.06 | 49.86 |
| Y ~ 1 | 25 | 8.49 | 2 | 0.01 | 73.41 |
| Y ~ Q | 25 | 11.48 | 4 | 0.00 | 29.95 |
| **Spring 2022 After Removing Single Malta Queen in Edmonton from the Data** | | | | | |
| Y ~ L | 24 | 0.00 | 3 | 0.72 | 31.28 |
| Y ~ 1 | 24 | 2.59 | 2 | 0.20 | 38.88 |
| Y ~ L + Q | 24 | 5.17 | 5 | 0.06 | 30.04 |
| Y ~ Q | 24 | 7.51 | 4 | 0.02 | 37.89 |
| Y ~ L * Q | 24 | 8.70 | 6 | 0.01 | 29.95 |
